# Characterization of the Complete Mitochondrial Genome of the Central Highland Grey-Shanked Douc Langur (*Pygathrix cinerea*), a Critically Endangered Species Endemic to Vietnam (Mammalia: Primates)

**DOI:** 10.3390/cimb46090592

**Published:** 2024-09-06

**Authors:** Mai Thi Phuong Nguyen, Tram Thi Thuy Nguyen, Tung Thanh Ha, Chi Nguyen Quynh Ho, Cuong Phan Minh Le, Huy Nghia Quang Hoang, Quynh Thi Nhu Nguyen, Tao Thien Nguyen, Dung Tri Luu, Khoa Dang Dang, Quan Ke Thai, Long Thanh Le

**Affiliations:** 1 Tay Nguyen Institute for Scientific Research, Academy of Science and Technology, Dalat City 670000, Vietnam; ntpmai@tni.vast.vn (M.T.P.N.); httung@yahoo.com (T.T.H.); 2 Biotechnology Department, Graduate University of Science and Technology, Vietnam Academy of Science and Technology, Hanoi 100000, Vietnam; tramttn@upes.edu.vn (T.T.T.N.); quynhchihonguyen@gmail.com (C.N.Q.H.); 3 Department of General and Biomedical Science, Ho Chi Minh City University of Physical Education and Sports, Ho Chi Minh 700000, Vietnam; dungtl@upes.edu.vn; 4 Animal Biotechnology Department, Institute of Tropical Biology, Vietnam Academy of Science and Technology, Ho Chi Minh 700000, Vietnam; lephanminhcuong@gmail.com (C.P.M.L.); hoangnghiaquanghuy@gmail.com (H.N.Q.H.); 5 Department of Bioactive Compounds, Institute of Tropical Biology, Vietnam Academy of Science and Technology, Ho Chi Minh 700000, Vietnam; quynhntn.itb@gmail.com; 6 Institute of Genome Research, Vietnam Academy of Science and Technology, Hanoi 100000, Vietnam; nguyenthientao@gmail.com; 7 Faculty of Biotechnology, Ho Chi Minh Open University, Ho Chi Minh 700000, Vietnam; dangdangkhoacm@gmail.com; 8 Faculty of Natural Sciences Education, Saigon University, Ho Chi Minh 700000, Vietnam; tkquan@sgu.edu.vn

**Keywords:** *Pygathrix cinerea*, Vietnam, mitogenome, phylogenetic

## Abstract

The grey-shanked douc langur (*Pygathrix cinerea*) is a recently described, critically endangered primate, endemic to Vietnam. In this study, we describe the Central Highland species’ complete mitochondrial genome (mitogenome—mtDNA). It is a circular molecule with a length of 16,541 base pairs (bp). The genome consists of 37 genes, consistent with those found in most other vertebrates, including 13 protein coding genes, 22 transfer RNAs, and two ribosomal RNAs. A comparison with the mitogenomes of more than 50 primates showed that the mitogenome of Vietnamese Central Highland *Pygathrix cinerea* has a conservative gene order. We identified 43 nucleotide differences when comparing this genome with a previously published mitogenome of *Pygathrix cinerea*. It is evident that there are distinct differences between the *Pygathrix cinerea* we are currently studying and other *Pygathrix cinerea* specimens. These differences are unlikely to be solely the result of sequencing errors, as the mitogenomes were generated using high-quality methods. The genetic divergence observed between the two *Pygathrix cinerea* mitogenomes implies the potential existence of at least two distinct lineages or forms of this primate species within its native range in Vietnam.

## 1. Introduction

The grey-shanked douc langur *(Pygathrix cinerea*) is a critically endangered primate species endemic to Vietnam. It was initially described as a subspecies of the Indochinese douc langur (*Pygathrix nemaeus*) in 1997 but subsequent morphological and molecular studies have confirmed it as a distinct species within the genus *Pygathrix* [1,2,3,4]. The grey-shanked douc langur is one of the rarest primates in the world, with a highly fragmented population estimated at fewer than 1000 individuals [5,6,7,8]. At present, its distribution is primarily within the Vietnamese provinces of Quang Nam, Quang Ngai, Kon Tum, and Gia Lai, inhabiting a limited range of tropical forests [8]. Morphological variability within the species has been observed, with some populations exhibiting distinct phenotypic characteristics whereby coat coloration can range from light grey to dark charcoal, with some individuals exhibiting reddish-brown hues on the limbs and head [2,3]. The grey-shanked douc langur faces significant threats to its long-term survival. Its population is projected to decline substantially between 2050 and 2070, primarily due to habitat loss and fragmentation driven by deforestation, infrastructure development, and the expansion of agriculture [9]. Conservation efforts are critical to prevent the extinction of this endangered primate. Understanding the genetic diversity and evolutionary history of the grey-shanked douc langur is essential for effective conservation planning and management [10,11,12,13,14]. Analyzing the mitochondrial genome can determine the origins of and genetic relationships between populations of a species. Populations with low genetic diversity will need to be prioritized for conservation to maintain the vitality of the species. This helps better understand the genetic structure and evolutionary history of the species, allowing for more effective conservation strategies [3,15,16,17,18,19,20,21,22]. We sequenced the complete mitochondrial genome of a grey-shanked douc langur from Vietnam and compared it to available mtDNA sequences from other douc langur species. This study presents the first complete mitochondrial genome sequence of *Pygathrix cinerea*, a critically endangered primate endemic to Vietnam. While partial mitogenome sequences of this species were included in Liedigk et al. [4], a detailed analysis of its mitochondrial characteristics was lacking. We compared the complete mitogenome of *Pygathrix cinerea* to publicly available data from other primates, focusing on the precise location and orientation of genes within the mitochondrion. Our analysis revealed that the gene order and orientation in the *Pygathrix cinerea* mitogenome are generally conserved among douc langur species (*Pygathrix*). However, we identified significant differences in the positions and lengths of a few genes, highlighting potential variations in mitochondrial evolution within this group. Phylogenetic analysis was conducted to assess the evolutionary relationships among these species. The results of this study provide valuable information for understanding the characteristics, the evolutionary history, and conservation status of the *Pygathrix cinerea*, a critically endangered species.

## 2. Materials and Methods

### 2.1. DNA Extraction and Sequencing

DNA Extraction: Vietnamese Central Highland *Pygathrix cinerea* tissue (Supplementary Figure S1) was collected in the Biological Museum, Tay Nguyen Institute for Scientific Research, Vietnam Academy of Science and Technology. Its mitogenome was extracted using the QIAamp DNA Mini kit (Qiagen, Germantown, MD, USA) following the manufacturer’s instructions. Evaluation of DNA sample quality: The DNA concentration was determined using the fluorometric method (Qubit). The OD_260_/OD_280_ ratio was determined by absorbance measurement. The DNA size was determined by agarose gel electrophoresis. Samples meeting the quality criteria of concentration ≥2 ng/µL, amount ≥90 ng, and OD_260_/OD_280_ ≥ 1.70 were considered suitable for further experimental steps. Samples with DNA size <1000 bp were flagged.

Sequencing: Whole genome sequencing libraries were prepared using the NEBNext Ultra II DNA Library Prep Kit for Illumina (New England Biolabs, Ipswich, MA, USA) following the manufacturer’s instructions. The library concentration was determined by the fluorometric method and the average library size was determined using a Bioanalyzer (Agilent, Santa Clara, CA, USA) according to Illumina’s library evaluation guidelines. Samples were considered suitable for sequencing when the concentration was ≥0.50 ng/µL (for genome size <1 Gb) or ≥2 ng/µL (for genome size >1 Gb). The libraries were then sequenced using the 150 PE Next Generation Sequencing method on a MiniSeq, MiSeq, or NovaSeq instrument (Illumina, San Diego, CA, USA) [23].

### 2.2. De Novo Assembly

De novo assembly is a method of constructing genome sequences from short or long reads without the need for them to be based on a reference genome sequence. We used the GetOrganelle (v1.7.7.0) pipeline with optimal parameters for the de novo assembly of the sample genome sequence. The assembly results are presented in Supplementary Table S4, showing a contig length of 16,656 bp consistent with the size of the mitochondrial genome, which was approximately 16 kb.

### 2.3. Bioinformatics Analysis

The raw sequencing data were purified using the fastp tool v0.23.1 [21]. Nucleotides with poor solution quality or that were unreliable or unknown (type N nucleotides) were eliminated based on the Phred-score value recorded for each nucleotide [22]. After being “purified”, the reads were assembled de novo using the GetOrganelle tool (v1.7.7.0) [24]. The raw sequencing data, after undergoing optimization, de novo assembly, and genome annotation, were stored in the FASTQ file format (Supplementary Data S2), containing read and sequence information and the corresponding quality score. The reading quality results are presented in Supplementary Table S3. The de novo assembly quality was assessed using the Quast v5.2.0 tool [22] and local alignment of reads onto assembled contigs. This allowed the detection of regions with unusually low depth coverage values compared to neighboring regions, which were noted in the assembly results. The assembly was annotated using the MITOS annotation system (revision 6b33f95) [25] with a specialized mitochondria database.

The mitogenome was annotated using Mitos WebServer version 2.1.9 [25] and Mito Fish version 4.03 [26]. Mito Annotator version 4.03 [26] was applied to create a genetic map of the entire *Pygathrix cinerea* mitogenome. Amino acid and nucleotide compositions were evaluated and compared for all 55 primate mitogenomes using MEGA 11: AT-skew = (A − T)/(A + T) and GC-skew = (G − T)/(G + T). The t-RNA sequences were aligned with the homologues of similar species. By comparing it to other *Pygathrix* mitogenomes, the A + T rich, PCGs, and r-RNAs were determined. The Relative Synonymous Codon Usage (RSCU) values of *Pygathrix cinerea*’s whole mitogenome were computed using MEGA 11. The secondary structures of transfer RNA (t-RNA) predictions were determined using tRNAscan-SE software v2.0 [27] and the Mitos WebServer [25].

### 2.4. Phylogenetic Tree

To determine the molecular location in the evolutionary tree of *Pygathrix cinerea* and its association with other primates, 13 PCGs of these 55 species’ sequence alignments were subjected to analysis with the Maximum Likelihood method using IQ-tree version 2.2.2.6 [28]. The appropriate substitution model for the data set was selected using ModelFinder version 2.2.0 [29]. Branch support was obtained using ultra-fast bootstrapping [30]. The GenBank accession numbers of all sequences used are shown in the Supplementary Materials.

## 3. Results and Discussion

### 3.1. Complete Mitochondrial Genome Analysis

**Figure 1:**
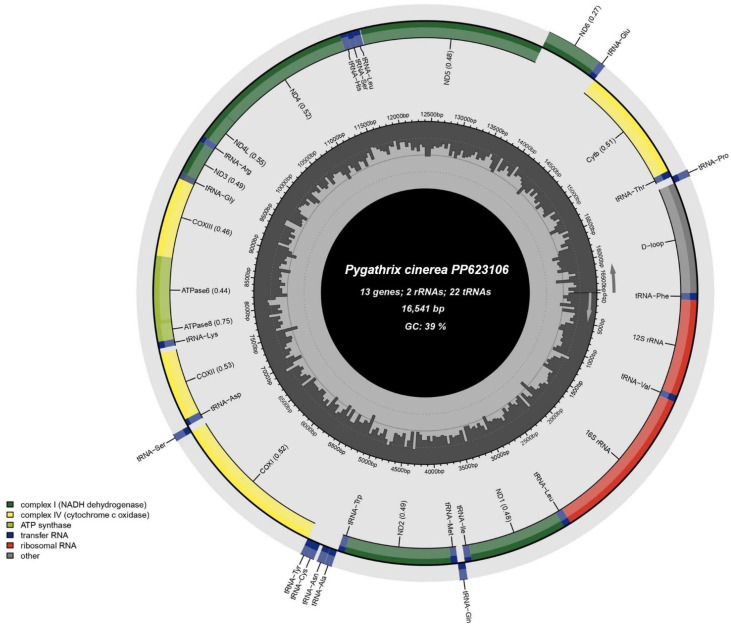
Genome structure of the complete mitochondrial of *Pygathrix cinerea*. The circular mitochondrial genome is depicted, with genes represented by colored blocks. The control region is shown in grey, the *16S* (large rRNA) and *12S* (small rRNA) genes are shown in red, the 22 transfer RNA (tRNA) genes are labeled in dark blue, and the 13 protein-coding genes (PCGs) are shown in green and yellow. Genes located on the H strand are shown on the outer circle, while genes located on the L strand are shown on the inner circle.

The complete mitochondrial genome of *Pygathrix cinerea* comprises 16,541 base pairs (bp) and harbors the typical 37 genes found in primate mitogenomes (Figure 1 and Table 1). These genes include a control region, two ribosomal RNA (rRNA) genes (*12S* rRNA and *16S* rRNA), 22 transfer RNA (tRNA) genes, and 13 protein-coding genes (PCGs). The majority of genes are located on the H strand, with the exception of ND6 and eight tRNA genes (tRNA-*Gln*, tRNA-*Ala*, tRNA-*Asn*, tRNA-*Cys*, tRNA-*Tyr*, tRNA-*Ser*, tRNA-*Glu*) residing on the L strand. The *Pygathrix cinerea* mitogenome exhibits a gene organization similar to that of other douc langur species. However, it is noteworthy that the complete mtDNA sequence of the Vietnamese Central Highland *Pygathrix cinerea* analyzed in this study is slightly larger (16,541 bp) than previously published sequences, including *Pygathrix cinerea* (JQ821842, 16,535 bp) and *Pygathrix nigripes* (MH064177, 16,536 bp) [4].

### 3.2. Nucleotide Composition Pattern

**Figure 2:**
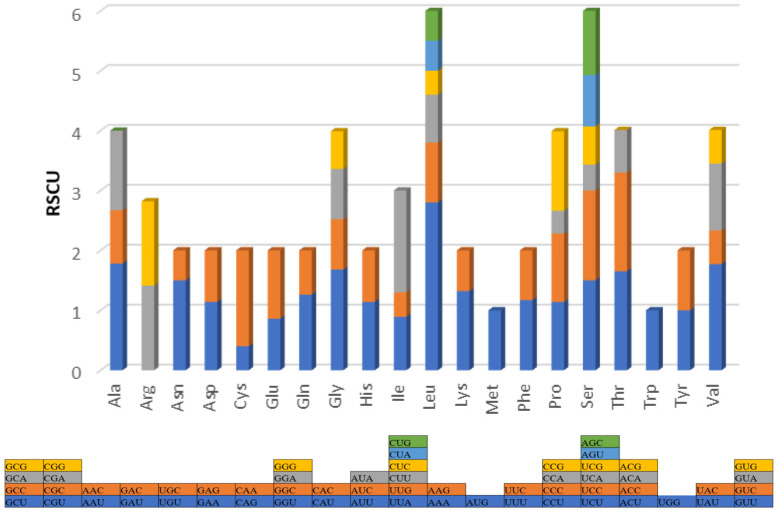
*Pygathrix cinerea* mitochondrial protein-coding genes’ Relative Synonymous Codon Usage (RSCU).

**Figure 3:**
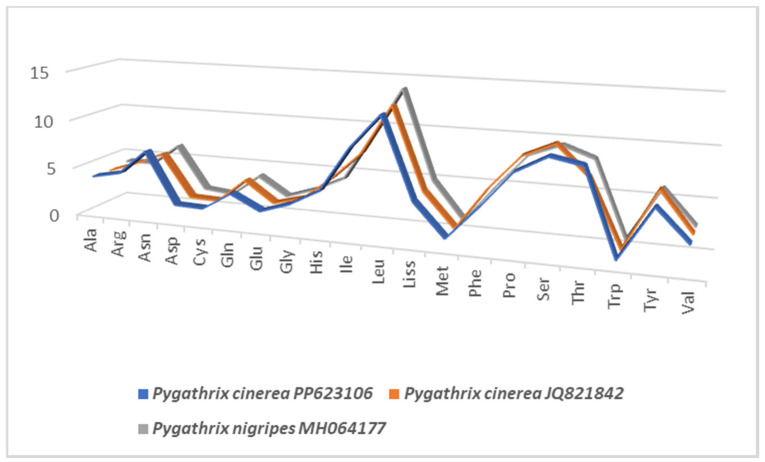
Percentage frequency of amino acid composition in the whole mitogenome of *Pygathrix* species: *Pygathrix cinerea* PP623106, *Pygathrix cinerea* JQ821842, and *Pygathrix nigripes* MH064177.

The Vietnamese Central Highland *Pygathrix cinerea* mitochondrial genome exhibits a high A + T content, accounting for 61.4%, which is similar to *Pygathrix nigripes* (61.1%) but differs slightly from other *Pygathrix cinerea* specimens (61.1%) and *Pygathrix nemaeus* (61.3%) (Table 2). This A + T bias is particularly pronounced in the *trnF* and *trnL1* genes, reaching 71%. Consistent with other primates, *Pygathrix cinerea* mtDNA displays a strong A/T preference in codon usage (Figure 2). Comparative analysis of amino acid frequencies across the three *Pygathrix* species reveals a highly similar distribution, as evidenced by comparable A + T content, AT-skew, and GC-skew (Figure 3). These metrics, commonly used to assess nucleotide composition patterns in mitochondrial genomes, highlight the conserved nature of amino acid frequencies within the *Pygathrix* genus. The most frequent amino acids in Vietnamese Central Highland *Pygathrix cinerea* and other *Pygathrix cinerea* specimens include *Ala* (3.9–4%), *Asp* (1.8%), *Cys* (1.7%), *Asn* (6.1–7.1%), *Gln* (3.5–4.1%), *Ile* (7.9–9.1%), *Phe* (4.4–5.4%), and *Thr* (7.3–9.1%).

### 3.3. Protein-Coding Genes

The *Pygathrix cinerea* mitogenome harbors the 13 canonical protein-coding genes (PCGs), collectively spanning 11,292 bp and representing 68.27% of the mitochondrial genome. Twelve of these PCGs reside on the H-strand (majority strand), while *nad6* is located on the L-strand (minority strand). The overall A + T content of the PCGs is 61.4%, ranging from 58.7% (*cox3*) to 68.9% (*atp8*). This A + T bias is consistent with the overall nucleotide composition of the *Pygathrix cinerea* mitogenome.

**Figure 4:**
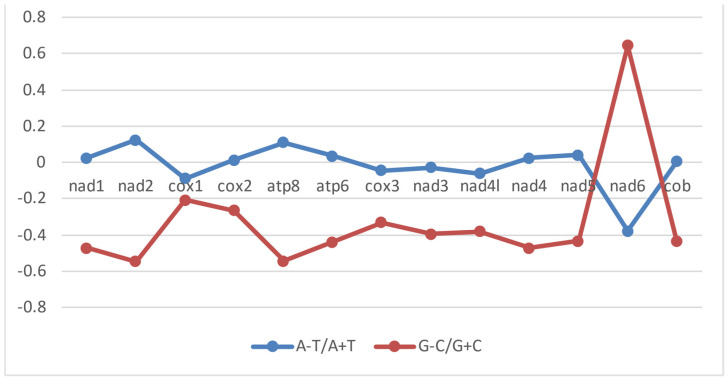
Graphical representation of AT- and GC-skew in all 13 protein-coding genes of *Pygathrix cinerea* PP623106 mitogenome.

To assess nucleotide composition bias across the PCGs, we calculated AT- and GC-skews. The majority of *Pygathrix cinerea* PCGs exhibit negative AT-skewness (Table 3), indicating a higher frequency of thymines than adenines. This pattern is also observed in other primates, where the majority of PCGs display negative GC-skewness, ranging from −0.35 to −0.42, suggesting a C-biased nucleotide composition. *nad6*, however, deviates from this trend, exhibiting a positive GC-skew (0.644) and a negative AT-skew (−0.378). This pattern is similar to that observed in other *Pygathrix cinerea* (−0.387 and 0.648) and *Pygathrix nigripes* (−0.341 and 0.596) samples (Figure 4).

All 13 PCGs in *Pygathrix cinerea* initiate with ATN codons (ATG or ATT), consistent with other *Pygathrix* species, However, *cob-1* in *Pygathrix nigripes* exhibits a distinct start codon, TTG, suggesting potential variation in translation initiation mechanisms within the *Pygathrix* genus.

### 3.4. A + T-Rich Region

The control region (D-loop), located between the *trn*P gene and *trn*F gene, spans 1094 base pairs (positions 15,584–16,541 and continuing at 1–136). This size is typical for vertebrate mitochondrial genomes [31].

The size of this region in Vietnamese Central Highland *Pygathrix cinerea* is larger than in other *Pygathrix cinerea* specimens (1092 bp; 15,444–16,535 nt) and in *Pygathrix nigripes* (1092 bp; 15,443–16,534 nt), *Macacac mulatta* (1085 bp; 15,480–16,564), *Papio hamadryas* (1076 bp; 15,446–16,521 nt), and *Callithrix jacchus* (1079 bp; 15,421–16,499 nt), but shorter than in *Homo sapiens* (1121 bp; 16,022–16,567nt, 1–575 nt). This size variation is primarily attributed to differences in copy numbers and tandem repeats within the region [32].

The control region exhibits a high degree of variation compared to other areas of the mitochondrial genome, reflecting the presence of multiple tandem repeats (TRs) and variations in their copy numbers [32]. The total GC-skew in the control region was −0.349, AT-skew was 58%, and A + T content was −0.013. No discernible repeat deletions were observed in the control region of Vietnamese Central Highland *Pygathrix cinerea*.

A comparison with other primate species revealed over 80 mutations between *Pygathrix cinerea* and *Pygathrix nigripes* within the control region. Most mutations were similar between different *Pygathrix cinerea* samples, except for nine positions (highlighted in orange in Table 4). These nine nucleotide mutations were either deletions or insertions, and they were unique to Vietnamese Central Highland *Pygathrix cinerea* and other *Pygathrix cinerea* specimens.

The short sequence (TATAA) was identified within the control region, occurring four times in *Pygathrix cinerea* and twice in other species. The sequence (AATAAT) also occurred twice in other *Pygathrix cinerea* specimens but was not present in *Pygathrix nigripes*. Additionally, the control region contains a high AT content (AATTATATAATCTATTA), which may represent a distinctive feature of Vietnamese Central Highland *Pygathrix cinerea*.

### 3.5. tRNA and rRNA Genes

**Figure 5:**
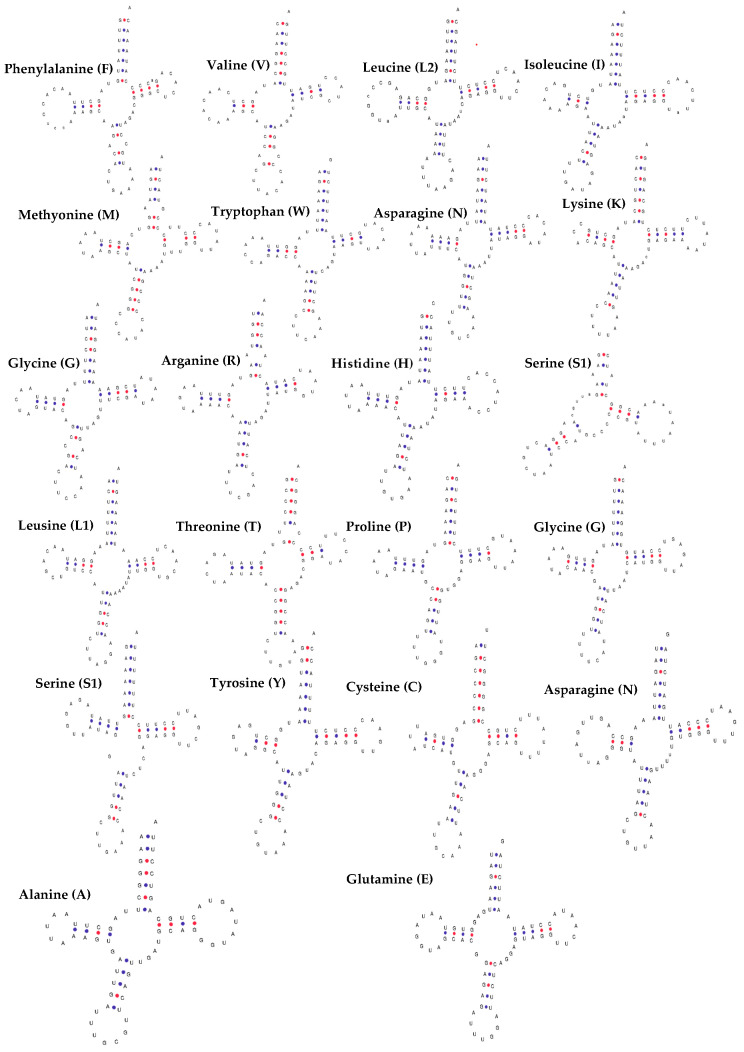
Secondary structure of 22 tRNA genes of Vietnamese Highland *Pygathrix cinerea*. Red color is GC connection, blue color is AU connection.

As predicted by Mitos WebServer, all 22 transfer RNA (tRNA) genes could be folded into a secondary clover-leaf structure (Figure 5). Eight tRNA genes were located on the L strand, while the remaining genes were located on the H strand (Table 1). All 22 tRNA genes identified in the *Pygathrix cinerea* mitochondrial genome have anticodons that are consistent with those found in other primates. The tRNA genes range in size from 59 bp (for *trnS1*) to 75 bp (for *trnL2*). The average base composition of the tRNA genes is A: 32.7%, T: 30.4%, G: 19.3%, and C: 17.5%. *trnT* has the highest GC content (50.8%), while *trnD* and *trnH* have the lowest (23.2%). In addition to the typical clover-leaf structure, seven tRNA genes in *Pygathrix cinerea* contain a total of eleven mismatched base pairs. These mismatches are found in the amino acid acceptor (AA) stem, the pseudouridine (TΨC) stem, and the anticodon (AC) stem (Table 5).

The mitochondrial genome of *Pygathrix cinerea* also contains two ribosomal RNA (rRNA) genes: the large ribosomal subunit (*16S* rRNA) and the small ribosomal subunit (*12S* rRNA). The *16S* rRNA gene is located between the *trnV* and *trnL2* genes, while the *12S* rRNA gene is located between the *trnF* and *trnV* genes. This arrangement is typical for most vertebrates, where the *trnV* gene separates the large and small ribosomal subunits [33]. The *12S* rRNA gene is 949 bp long, and the *16S* rRNA gene is 1565 bp long. These sizes are similar to those found in other *Pygathrix cinerea* specimens (948 bp and 1562 bp, respectively). The A + T content of the *16S* rRNA gene is 60%, and the A + T content of the *12S* rRNA gene is 59.4%. These values are consistent with those observed in other primates, including other *Pygathrix cinerea* specimens (59.9% for *16S* and 59.4% for *12S*) and *Pygathrix nigripes* (60% for *16S* and 58.8% for *12S*) (Table 2).

### 3.6. Intergenic Region and Overlapping

#### 3.6.1. Gene Overlaps

Comparative analysis of the mitochondrial genomes reveals several instances of gene overlaps:

*atp8* and *atp6*: a 22 bp overlap was consistently observed between the *atp8* and *atp6* genes in Vietnamese Central Highland *Pygathrix cinerea*, other *Pygathrix cinerea* specimens, and *Pygathrix nigripes*.
*trnV* and *rrnL*: a 2 bp overlap was found in Vietnamese Central Highland *Pygathrix cinerea*, other *Pygathrix cinerea* individuals, and *Pygathrix nigripes*.
*trnI* and *trnQ*: a 3 bp overlap was present in Vietnamese Central Highland *Pygathrix cinerea*, other *Pygathrix cinerea* individuals, and *Pygathrix nigripes*.
*nad4L* and *nad4*: a 4 bp overlap was observed in Vietnamese Central Highland *Pygathrix cinerea* and other *Pygathrix cinerea* specimens.
*cob-0* and *cob-1*: an 8 bp overlap was uniquely found in *Pygathrix nigripes*.

These overlaps, particularly the 22 bp overlap between *atp8* and *atp6* and the 2 bp overlap between *trnV* and *rrnL*, are common among *Pygathrix* species and are located on the H strand.

#### 3.6.2. Intergenic Spacer Regions

The *Pygathrix cinerea* mitochondrial genome contains approximately twenty non-coding intergenic spacer regions ranging in size from 1 bp to 68 bp (Table 1). While these regions were previously considered non-functional, recent studies suggest that some may play regulatory roles in gene expression.

The longest intergenic spacer in *Pygathrix cinerea* is 68 bp long, located between *trnK* and *cox2*. This spacer is significantly shorter than the 172 bp spacer found in other *Pygathrix cinerea* specimens and the 167 bp spacer found in *Pygathrix nigripes*.

Intergenic spacer lengths vary across *Pygathrix* species, with *Pygathrix cinerea* having shorter spacers than *Pygathrix nemaeus* (194 bp with 20 regions) and *Macaca leonnina* (182 bp with 22 regions) but longer spacers than *Pygathrix nigripes* (167 bp with 20 regions). The intergenic spacer regions between *Pygathrix cinerea* and *Pygathrix nigripes* are generally similar, except for the region between *nad5* and *trnL1*, which is 15 bp in *Pygathrix cinerea* and 37 bp in *Pygathrix nigripes*.

### 3.7. Mutations

While many of the positions differed between Vietnamese Central Highland *Pygathrix cinerea* and other *Pygathrix* species (Supplementary Table S5), only a few of them resulted in changes to the encoding of amino acids (Supplementary Table S6), which demonstrates the distinctive features of Vietnamese Central Highland *Pygathrix cinerea* compared to other species. In particular, 43 mutation positions in the DNA (Table 6) that encoded for 23 amino acids differed between Vietnamese Central Highland *Pygathrix cinerea* and other *Pygathrix cinerea* specimens *(*Table 7). Among them, four nucleotide insertion positions were found in *rrnS* and *rrnL*; others included 30 nucleotide substitution or deletion positions in PCG and nine nucleotide substitution or deletion positions in the control region. The insertion T at position 242 encodes for *rrnS* (208–1156); A at position 1711; C at position 2772; A at position 2773, leading to the insertion of one more amino acid leucine at the 81st position in *rrnS*; the amino acid isoleucine at position 571 in *rrnL*; the amino acid histidine at the 924th position in *rrnL*; and the amino acid asparagine at position 925 in *rrnL*, none of which were found in the other *Pygathrix cinerea* specimens. In addition, in the *rrnL* genome, the substitution mutation at the 2566th position also differed, from glycine in the other *Pygathrix cinerea* specimens to asparagine in Vietnamese Central Highland *Pygathrix cinerea*. Most PCGs exhibited substitutions or deletion mutations that led to changes in the amino acid which it codes. The deletion from C in other *Pygathrix cinerea* specimens in Vietnamese Central Highland *Pygathrix cinerea* at positions 3080 and 3134 resulted in changing threonine to isoleucine at the 1027th and 1045th positions. In contrast, at the 3296th position, nucleotide G, which changes lysine to arginine at position 1099th in *nad1*, occurs in Vietnamese Central Highland *Pygathrix cinerea* but not in other species.

In *nad2*, *trbY*, and *nad4*, there was one deletion in each PCG of the Vietnamese Central Highland *Pygathrix cinerea* mitogenome, which changed the protein that they code. More specifically, in *nad2*, *trbY*, and *nad4*, the deletion at positions 4997, 5414, and 10,519 derived isoleucine to threonine at positions 1666 and 1805, and cysteine to serine at position 3507 in Vietnamese Central Highland *Pygathrix cinerea*, respectively.

There were two deletion mutations in *cox1* at the 5507th and 5678th loci. In comparison with other *Pygathrix cinerea* specimens, at position 5507 in the Vietnamese Central Highland *Pygathrix cinerea* mitogenome, A was not seen, while C did not occur at the 5678th position in the other *Pygathrix cinerea* specimens, meaning that cysteine was present at position 1836 instead of tyrosine and serine was present at the 1893rd position instead of cysteine, respectively.

G was present at position 13,125 in Vietnamese Central Highland *Pygathrix cinerea* instead of the deletion found in other *Pygathrix cinerea* specimens, meaning that there was tryptophan instead of no amino acid at position 4375 in the *nad5* genome. At protein positions 413 and 414, which correspond to the 1206th base pair, there was still an insertion mutation.

In *nad6* at the 13,942nd and 13,957th loci, nucleotide T was found in Vietnamese Central Highland *Pygathrix cinerea*; however, it was not found in other samples. This led to the amino acids being present at different positions (4648th and 4653rd), replacing histidine with tyrosine and proline with serine.

More specially, three positions did not occur in the *cob* genome of Vietnamese Central Highland *Pygathrix cinerea* in comparison with other *Pygathrix cinerea* specimens. At position 14,749, G was deleted, and at positions 14,659 and 14,998, C was found. Therefore, there were three differences in the amino acids compared with other *Pygathrix cinerea* specimens in terms of *cob* protein: aspartic acid instead of cysteine at the 4917th position, phenylalanine instead of serine at the 4920th position, and tyrosine instead of histidine at the 5000th position.

### 3.8. Evolutionary Analysis

The Tamura–Nei model and the Maximum Likelihood approach were used to deduce the evolutionary history [34]. The branches show the proportion of trees where the related taxa are grouped together. The resulting tree had the highest log likelihood (−433,849.22). By applying the Neighbor-Join and BioNJ algorithms on a matrix of pairwise distances computed using the Tamura–Nei model, the initial tree(s) for the heuristic search were automatically created. The topology with the superior log likelihood value was then selected. This analysis included 55 mitogenomes. The codon positions included were 1st, 2nd, 3rd and Noncoding. Evolutionary analyses were conducted in MEGA11 [33].

### 3.9. Phylogeny of 55 Primate Mitogenomes

**Figure 6:**
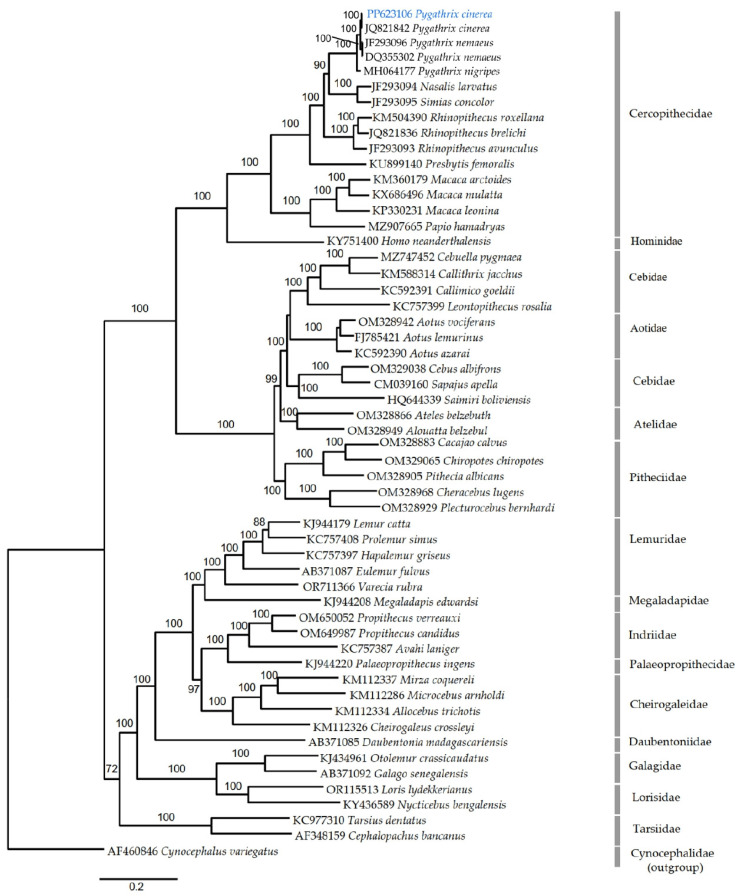
Maximum likelihood phylogeny of 55 whole primate mitogenomes. The PP633106 *Pygathrix cinerea* in blue color is the Vietnamese Central Highland *Pygathrix cinerea*.

The phylogeny of primates is shown in Figure 6. The tree topology of the primates was consistent according to the Maximum Likelihood method and analysis with bootstrap support (>70). The closest living relatives of primates are *Hominidae*. The tree was rooted with the outgroup *Cynocephalus variegatus* based on Mason et al. [35]. The entire Primates tree has its roots stemming from the species of Dermoptera, specifically *Cynocephalus variegatus*, and the resulting structure is consistent with the structure obtained from previous studies [36,37,38,39,40]. The phylogenetic tree constructed using the complete mitochondrial genomes of over 50 primate species clearly shows that the *Pygathrix* species (*Pygathrix cinerea*, *Pygathrix nemaeus*, and *Pygathrix nigripes*) form a well-supported monophyletic clade. In previous studies, the relative position of *Pygathrix* and *Nasalis* was closer than *Rhinopithecus*, and the present study revealed the same thing [2,4]. A common origin of the group is now widely accepted, although the phylogenetic relationships among its genera and species are largely unknown [4]. Our analysis strongly supports this relationship among primates and confirms that the douc langurs (genus *Pygathrix*) are a distinct evolutionary lineage within the odd-nosed monkey group. The *Pygathrix* clade is most closely related to the genera *Nasalis* (proboscis monkey) and *Simias* (*Mentawai langur*). Together, these three genera form a larger monophyletic group of odd-nosed monkeys, which is consistent with previous morphological and molecular studies.

The mitochondrial genome sequence of *Pygathrix cinerea* generated in this study (GenBank accession PP623106) is significantly different from a previously published *Pygathrix cinerea* mitogenome (GenBank accession JQ821842) with 43 nucleotide differences, suggesting the presence of distinct genetic lineages within this species. This divergence between the mitogenomes indicates the previously published sequence (JQ821842) may have come from a different geographic population or subspecies of *Pygathrix cinerea*, which could account for the observed genetic differences or the possibility of cryptic subspecies or evolutionarily significant units within the grey-shanked douc langur population. The mitogenome sequence generated in this study is firmly placed within the *Pygathrix* clade, confirming the species assignment of the sample as *Pygathrix cinerea*. However, the distinct genetic differences from the previously published *Pygathrix cinerea* mitogenome suggest the need for more population investigation into the evolutionary history and population structure of this critically endangered primate species.

## 4. Discussion

The mitochondrial genome of Vietnamese Central Highland *Pygathrix cinerea* reveals a fascinating blend of conserved features characteristic of primates and unique variations that offer insights into its evolution and conservation. Our analysis provides valuable data for understanding the genetic diversity within this threatened species and its potential implications for conservation efforts.

### 4.1. Control Region: A Window into Evolutionary History and Population Dynamics

The control region of Vietnamese Central Highland *Pygathrix cinerea* exhibits a significantly larger size (1094 bp) compared to other *Pygathrix cinerea* specimens (1092 bp); however, the two base pair difference is in a region of repetitive sequences and is therefore biologically insignificant, as this pattern is observed in other primate species that do not experience population bottlenecks or rapid evolutionary divergence [41]. Therefore, the minor size difference observed here does not warrant conclusions about distinct evolutionary lineages or population-level diversity.

This size variation, coupled with the presence of unique mutations, suggested a potentially distinct evolutionary lineage for this population, possibly reflecting a period of isolation or adaptation to specific environmental pressures [42]. These mutations might be associated with the effect of selection and genetic drift and supports the notion of a distinct evolutionary lineage [43]. However, the limited number of samples precludes any robust conclusions about the evolutionary history and population structure of this endangered primate.

The tandem repeats and GC-skew observed in the control region are consistent with other primates, highlighting the conserved nature of these regulatory elements. The specific arrangement and composition of these elements might influence replication initiation or transcription regulation [44].

### 4.2. Ribosomal RNA Genes: Essential for Protein Synthesis and Phylogenetic Resolution

The remarkable conservation of rRNA gene size and A + T content across *Pygathrix* species underscores their fundamental role in mitochondrial protein synthesis [45]. This consistency highlights the value of these genes as reliable markers for phylogenetic analysis, particularly for resolving relationships within closely related taxa [46,47]. While the rRNA genes are generally highly conserved, there are four nucleotide insertion positions found in *rrnS* and *rrnL* that may lead to regulate gene expression at the translational level and this rRNA modification pattern may occur in response to environmental shifts, developmental stages, and disease states [48] or indicate a unique evolutionary trajectory of Vietnamese Central Highland *Pygathrix cinerea*.

### 4.3. Transfer RNA Genes: Adapting to Specific Needs and Environmental Pressures

The presence of all 22 tRNA genes with consistent anticodons in *Pygathrix cinerea* is essential for the accurate translation of mitochondrial proteins. The conserved nature of these genes underscores their fundamental role in maintaining mitochondrial function. The variations in tRNA gene size and GC content, observed in Vietnamese Central Highland *Pygathrix cinerea*, could reflect differences in gene expression and regulation [49].

The identification of mismatched base pairs in several tRNA genes, particularly in the AA, TΨC, and AC stems, is intriguing. These mismatches could potentially influence tRNA structure and function.

### 4.4. Gene Overlaps and Intergenic Spacers: Unraveling Regulatory Mechanisms and Evolutionary Adaptations

The consistent gene overlaps observed between a pair of genes (*atp8* and *atp6*; *trnV* and *rrnL*) in *Pygathrix* species suggest a potential functional significance beyond mere chance occurrences. These overlaps might play a regulatory role in gene expression, potentially influencing the transcription or translation of these genes [50].

The intergenic spacer regions, while previously considered non-functional, are now recognized as potential regulatory elements [51]. The variations in spacer lengths across *Pygathrix* species, with longer spacers in *Pygathrix nemaeus* and *Macaca leonnina* compared to *Pygathrix cinerea*, suggest a potential role in regulating gene expression [52].

The unique genetic features identified in Vietnamese Central Highland *Pygathrix cinerea*, particularly in the control region and tRNA genes, highlight the importance of conserving its genetic diversity. This diversity is crucial for adaptation and resilience in the face of environmental changes and threats. Understanding the genetic differences between Vietnamese Central Highland *Pygathrix cinerea* and other *Pygathrix cinerea* specimens can inform conservation efforts by identifying distinct populations that may require specific management strategies.

Further research is needed to investigate the functional significance of the observed variations in the control region, tRNA genes, and intergenic spacer regions. Comparative studies with a broader range of primate species could provide a more comprehensive understanding of the evolutionary dynamics and functional implications of these variations. Additionally, examining the expression patterns of these genes in response to environmental stressors could shed light on their potential role in adaptation and resilience.

## 5. Conclusions

This study presents the first comprehensive characterization of the complete mitochondrial genome of the Central Highland grey-shanked douc langur, *Pygathrix cinerea*, a critically endangered primate endemic to Vietnam. The mitogenome sequence obtained provides valuable molecular data on this poorly studied species. The *Pygathrix cinerea* mitogenome is shown to retain most of the conserved genes of ancestral features. The mtDNA of this Vietnamese Central Highland *Pygathrix cinerea* consists of 37 genes that are typically similar to most vertebrates, arranged in a specific pattern. It is a circular molecule with a length of 16,541 base pairs (bp). These genes include 13 protein-coding genes, 22 transfer RNAs, and two ribosomal RNAs. This Vietnamese Central Highland *Pygathrix cinerea* is most closely related to *Pygathrix nemaeus* and the second closest relative is *Pygathrix nigripes*. Of particular note is that within the population of *Pygathrix cinerea*, there are significant differences in the gene structure with 43 distinct variations in the DNA that alter 23 amino acids in proteins, highlighting the differences between this Vietnamese Central Highland *Pygathrix cinerea* and other *Pygathrix cinerea*.

To better understand the genetic diversity, population dynamics, and conservation status of *Pygathrix cinerea*, future studies will need to incorporate a much larger sample size representing the major subpopulations across the species’ distribution. Comprehensive population genomic analyses incorporating nuclear as well as mitochondrial markers will be crucial to elucidate the evolutionary history and guide evidence-based conservation efforts for this threatened primate.

The mitogenome sequence reported here serves as an initial molecular resource, but additional research is needed to fully characterize the genetic diversity and evolutionary patterns within the grey-shanked douc langur. Expanding the genomic sampling and integrating it with ecological and demographic data will be necessary to inform effective conservation strategies for protecting this endangered species.

## Figures and Tables

**Table 1:** Location and length of 37 genes in the complete mitochondrial genome of *Pygathrix cinerea*.

Name	Start	Stop	Strand	Length	Intergenic Nucleotides
*trnF*(ttc)	137	207	+	71	0
*rrnS*	208	1156	+	949	0
*trnV*(gta)	1157	1222	+	66	−2
*rrnL*	1221	2785	+	1565	0
*trnL2*(tta)	2786	2860	+	75	2
*nad1*	2863	3813	+	951	4
*trnI*(atc)	3818	3887	+	70	−3
*trnQ*(caa)	3885	3956	-	72	0
*trnM*(atg)	3957	4024	+	68	0
*nad2*	4025	5062	+	1038	4
*trnW*(tga)	5067	5133	+	67	7
*trnA*(gca)	5141	5209	-	69	1
*trnN*(aac)	5211	5283	-	73	32
*trnC*(tgc)	5316	5381	-	66	0
*trnY*(tac)	5382	5446	-	65	1
*cox1*	5448	6989	+	1542	0
*trnS2*(tca)	6990	7058	-	69	3
*trnD*(gac)	7062	7130	+	69	1
*cox2*	7132	7791	+	660	68
*trnK*(aaa)	7860	7927	+	68	1
*atp8*	7929	8111	+	183	−22
*atp6*	8090	8764	+	675	5
*cox3*	8770	9552	+	783	1
*trnG*(gga)	9554	9620	+	67	0
*nad3*	9621	9965	+	345	1
*trnR*(cga)	9967	10,031	+	65	0
*nad4l*	10,032	10,325	+	294	−4
*nad4*	10,322	11,689	+	1368	10
*trnH*(cac)	11,700	11,768	+	69	0
*trnS1*(agc)	11,769	11,827	+	59	0
*trnL1*(cta)	11,828	11,898	+	71	3
*nad5*	11,902	13,701	+	1800	15
*nad6*	13,717	14,235	-	519	0
*trnE*(gaa)	14,236	14,304	-	69	4
*Cob*	14,309	15,442	+	1134	7
*trnT*(aca)	15,450	15,514	+	65	2
*trnP*(cca)	15,517	15,583	-	67	

**Table 2:** Nucleotide composition indices in various regions of twenty-three representative primate mitogenomes.

	Accession Number	Whole		Protein-Coding Genes (PCGs)		Large Ribosomal RNA (*rrnL*)		Small Ribosomal RNA (*rrnS*)	
		Length	AT (%)	Length (bp)	AT (%)	Length (bp)	AT (%)	Length (bp)	AT (%)
Vietnamese Central Highland *Pygathrix cinerea*	PP623106	16,541	61.1	11,292	61	1565	60	949	59.4
*Pygathrix cinerea*	JQ821842	16,535	61.1	11,292	61	1562	59.9	948	59.4
*Pygathrix nemaeus*	JF293096	15,467	61.3	11,292	61	1564	59.7	948	59.4
*Pygathrix nigripes*	MH064177	16,534	61.4	11,304	62	1563	59.7	949	58.8
*Macaca leonina*	KP330231	17,050	57.2	11,310	57	1562	57.8	947	54.6
*Presbytis femoralis*	KU899140	16,548	61.9	11,277	62	1563	61	935	58.3
*Nasalis larvatus*	DQ355298	16,570	60.9	11,298	61	1568	59.9	949	57.5
*Rhinopithecus roxellana*	KM504390	16,552	61.5	11,292	62	722	59.6	949	58.2
*Rhinopithecus brelichi*	JQ821836	16,553	61.6	11,295	62	1571	60	949	58.3
*Rhinopithecus bieti*	JQ821839	16,550	61.5	11,298	62	1570	59.7	949	58
*Rhinopithecus avunculus*	JF293093	16,552	61.6	11,289	62	1572	60.2	949	58.5
*Macaca arctoides*	KM360179	16,559	56.7	11,313	56	1566	57.3	951	55.8
*Macaca mulatta*	JQ821843	16,564	56.8	11,310	56	1560	57.4	947	55.6
*Papio hamadryas*	NC001992	16,521	56.3	11,292	56	1572	57.7	947	55.1
*Callithrix jacchus*	KM588314	16,499	59.7	11,280	59	1555	60.5	953	56.6
*Homo sapiens neanderthalensis*	NC011137	16,565	55.6	11,283	55	1560	57.2	954	54.4
*Homo sapiens neanderthalens*	OM062614	16,565	55.7	11,283	55	1560	57.3	954	54.4
*Cebus albifrons*	NC002763	16,554	60.9	11,268	61	1556	60.2	958	57.6
*Moschiola indica*	NC037993	16,444	61.4	11,310	61	1576	61.6	958	56.4

**Table 3:** GC-skew and AT-skew in PCGs.

	Accession Number	T(U)	C	A	G	Total	AT-Skew	GC-Skew
Vietnamese Central Highland *Pygathrix cinerea*	PP623106	30.9	26.1	30.5	12.5	868.6	−0.00605	−0.35278
*Pygathrix cinerea*	JQ821842	30.9	26.1	30.5	12.5	868.6	−0.0062	−0.35277
*Pygathrix nemaeus*	JF293096	30.9	26.1	30.5	12.5	868.6	−0.00678	−0.35155
*Pygathrix nigripes*	MH064177	31.2	25.9	30.6	12.3	807.4	−0.01002	−0.35463
*Macaca leonina*	KP330231	26.5	30.9	30.2	12.3	870	0.065732	−0.42945
*Presbytis femoralis*	KU899140	31.5	25.4	30.9	12.2	867.5	−0.00896	−0.34998
*Nasalis larvatus*	DQ355298	30.5	26.7	30.6	12.2	869.1	0.001593	−0.37082
*Rhinopithecus roxellana*	KM504390	31.4	25.6	30.8	12.2	868.6	−0.01054	−0.35253
*Rhinopithecus brelichi*	JQ821836	31.6	25.5	30.6	12.4	868.8	−0.01638	−0.34612
*Rhinopithecus bieti*	JQ821839	31.5	25.6	30.7	12.2	869.1	−0.01181	−0.35612
*Rhinopithecus avunculus*	JF293093	31.5	25.5	30.8	12.3	868.4	−0.01138	−0.34898
*Macaca arctoides*	KM360179	26.4	31	30	12.5	870.2	0.063577	−0.4244
*Macaca mulatta*	JQ821843	26.6	31	29.8	12.6	870	0.056444	−0.42052
*Papio hamadryas*	NC001992	26.2	31.2	29.7	12.9	868.6	0.061768	−0.41663
*Callithrix jacchus*	KM588314	28.3	27.8	31.1	12.9	867.7	0.046296	−0.36736
*Homo sapiens neanderthalensis*	OM062614	26.2	31.9	28.8	13.1	867.8	0.046422	−0.41631
*Cebus albifrons*	NC002763	29.9	26.7	31.3	12.1	866.8	0.02306	−0.37663

**Table 4:** Different positions in control region of Vietnamese Central Highland *Pygathrix cinerea*, other *Pygathrix cinerea* specimens, and *Pygathrix nigripes*. The letters A, T, G, and C represent the four nucleotide bases in mtDNA: **A** (Adenine) **T** (Thymine) **G** (Guanine) **C** (Cytosine). The missing nucleotide base are represent in “.”.

**Position**	**1**	**1**	**1**	**1**	**1**	**1**	**2**	**2**	**2**	**2**	**2**	**3**	**3**	**3**	**3**	**3**	**3**	**3**	**3**	**3**	**3**	**3**	**3**	**3**	**3**
	**4**	**5**	**6**	**6**	**8**	**8**	**5**	**5**	**8**	**9**	**9**	**1**	**0**	**1**	**2**	**2**	**3**	**3**	**3**	**3**	**3**	**4**	**4**	**5**	**7**
	**7**	**8**	**1**	**9**	**0**	**4**	**0**	**8**	**9**	**0**	**4**	**7**	**5**	**5**	**6**	**7**	**0**	**2**	**3**	**6**	**7**	**1**	**5**	**8**	**9**
*Pygathrix nigripes* MH064177	G	C	A	C	T	C	G	T	C	A	A	C	A	C	C	T	C	G	A	C	T	A	G	C	C
*Pygathrix cinerea* JQ821842	A	T	G	T	C	T	A	C	T	G	G	A	G	T	T	C	A	A	G	T	A	G	A	T	T
*Pygathrix cinerea* PP623106	A	T	G	T	C	T	A	C	T	G	G	A	G	T	T	C	A	A	G	T	A	G	A	T	T
**Position**	**3**	**3**	**3**	**3**	**3**	**3**	**3**	**3**	**3**	**3**	**3**	**3**	**3**	**4**	**4**	**4**	**4**	**4**	**4**	**4**	**4**	**4**	**5**	**5**	**5**
	**6**	**6**	**6**	**6**	**6**	**7**	**7**	**7**	**8**	**8**	**8**	**8**	**9**	**1**	**4**	**5**	**6**	**6**	**6**	**6**	**8**	**9**	**0**	**0**	**2**
	**2**	**3**	**5**	**6**	**8**	**3**	**5**	**6**	**1**	**2**	**4**	**9**	**0**	**6**	**8**	**1**	**3**	**4**	**7**	**9**	**3**	**0**	**6**	**9**	**8**
*Pygathrix nigripes* MH064177	A	G	T	A	C	T	T	A	T	C	G	C	C	T	T	T	T	A	A	T	T	C	A	T	G
*Pygathrix cinerea* JQ821842	T	A	A	C	T	C	C	.	C	A	A	T	T	C	C	G	C	G	G	C	C	T	T	C	A
Vietnamese Central Highland *Pygathrix cinerea* PP623106	C	A	A	T	.	C	C	G	C	A	A	T	T	C	C	G	C	G	G	C	C	T	T	C	.
**Position**																				**1**	**1**	**1**	**1**	**1**	
	**6**	**6**	**6**	**6**	**6**	**6**	**8**	**8**	**8**	**8**	**9**	**9**	**9**	**9**	**9**	**9**	**9**	**9**	**9**	**0**	**0**	**0**	**0**	**0**	
	**1**	**1**	**1**	**1**	**5**	**5**	**1**	**5**	**7**	**8**	**3**	**3**	**4**	**4**	**4**	**5**	**6**	**7**	**8**	**0**	**1**	**1**	**2**	**6**	
	**1**	**3**	**5**	**9**	**3**	**7**	**4**	**0**	**9**	**3**	**4**	**6**	**2**	**4**	**7**	**8**	**0**	**0**	**0**	**3**	**1**	**4**	**7**	**5**	
*Pygathrix nigripes* MH064177	T	T	T	A	G	T	A	T	C	C	T	C	C	T	A	C	A	T	A	T	C	C	T	A	
*Pygathrix cinerea* JQ821842	C	.	C	G	A	C	G	C	T	.	C	T	T	A	G	T	G	C	G	C	T	.	A	G	
Vietnamese Central Highland *Pygathrix cinerea* PP623106	C	C	C	G	A	C	G	C	T	T	C	T	T	A	G	T	G	C	.	C	T	T	A	G	

**Table 5:** *Pygathrix cinerea*. The mismatched *t-RNAs* base pairs from *Pygathrix cinerea*. AA—amino acid acceptor, T-arm, AC—anticodon.

tRNA	Mismatched Base Pairs	Stem	Frequency
Phenylalanine GAA	A-C	AC	1
Valine TAC	A-C	AC	1
Methionine CAT	A-G	AA	1
	U-U	T-arm	2
	C-U	T-arm	1
	A-U	T-arm	1
Glycine TCC	A-C	AC	1
Serine GCT	A-A	AA	1
	A-A	AC	1
Threonine TGT	U-C	AA	1

**Table 6:** Mutation of DNA in whole genome between Vietnamese Central Highland *Pygathrix cinerea* and other *Pygathrix cinerea* specimens. Dot “.” indicates nucleotide deletion; “-” indicates nucleotide insertion.

Location																							1	1	1	1	1	1	1	1	1	1	1	1	1	1	1	1	1	1	1	1	1
		1	1	2	2	2	3	3	3	3	4	4	4	4	5	5	5	5	6	7	7	8	0	0	2	3	3	3	4	4	4	5	5	5	5	5	5	5	5	6	6	6	6
	2	2	7	5	7	7	0	1	2	9	4	6	8	9	0	4	5	6	6	0	5	9	3	5	8	1	9	9	7	7	9	0	3	5	8	8	8	8	9	0	3	4	4
	4	0	1	6	7	7	8	3	9	0	0	8	8	9	6	1	0	7	6	8	0	1	6	1	3	2	4	5	4	5	9	2	8	1	1	2	2	3	8	6	3	3	6
	2	6	1	6	2	3	0	4	6	6	7	9	7	7	4	4	7	8	3	3	9	7	0	9	7	5	2	7	9	9	8	7	2	1	6	0	2	0	2	7	7	4	8
*Pygathrix cinerea* NC 018063.1	-	A	-	G	-	-	C	C	.	.	T	.	C	T	T	T	A	.	.	A	.	T	G	C	G	.	.	.	G	C	C	T	C	T	T	C	T	.	A	.	.	G	.
Vietnamese Central Highland *Pygathrix cinerea* PP623106	T	.	A	.	C	A	.	.	G	T	.	C	.	.	.	.	.	C	G	A	T	.	.	.	.	G	T	T	.	.	.	.	.	T	C	T	.	G	.	C	T	.	T

**Table 7:** Mutation of protein in whole genome between Vietnamese Central Highland *Pygathrix cinerea* and other *Pygathrix cinerea* specimens. Dot “.” indicates nucleotide deletion; “-” indicates nucleotide insertion.

Location						1	1	1	1	1	1	1	3	4	4	4	4	4	5	5	5	5	5
		5	8	9	9	0	0	0	6	8	8	8	5	3	6	6	9	9	0	3	3	4	4
	8	7	5	2	2	2	4	9	6	0	3	9	0	7	4	5	1	2	0	2	5	4	9
	1	1	6	4	5	7	5	9	6	5	6	3	7	5	8	3	7	0	0	8	6	6	0
*Pygathrix cinerea* NC 018063.1	-	-	D	-	-	T	S	.	I	I	Y	.	P	.	.	.	D	S	H	N	.	.	.
Vietnamese Central Highland *Pygathrix cinerea* PP623106	L	I	.	H	N	.	.	R	.	.	.	S	.	W	Y	S	.	.	.	.	T	I	S

## Data Availability

Data are contained within the article and Supplementary Materials.

## Supplementary Materials

The following supporting information can be downloaded at: https://www.mdpi.com/article/10.3390/cimb46090592/s1. Figure S1: Vietnamese Central Highland *Pygathrix cinerea*; Data S2: Complete mtDNA genome of Vietnamese Central Highland *Pygathrix cinerea*; Table S3: Read quality (illumina) of analyzed samples; Table S4: De novo assembly result; Table S5: Mutation between three *Pygathrixes*; Table S6: Protein mutation between two *Pygathrixes*.
