# Supplementary material for: Characterization of the Complete Mitochondrial Genome of the Central Highland Grey-Shanked Douc Langur (Pygathrix cinerea), a Critically Endangered Species Endemic to Vietnam (Mammalia: Primates)
Source: Curr Issues Mol Biol. 2024 Sep 6;46(9):9928–47. doi: 10.3390/cimb46090592 (PMC11430490; doi:10.3390/cimb46090592)
Supplement: Supplementary file 1 [file cimb-46-00592-s001.zip › Supplementary data 1.pdf]

Supplementary 1: Vietnamese Pygathrix Cinerea

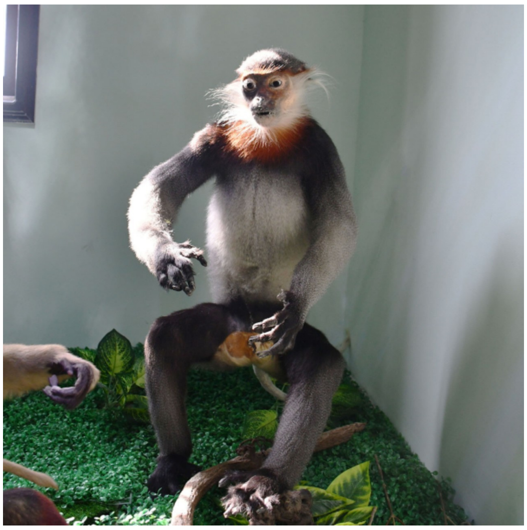

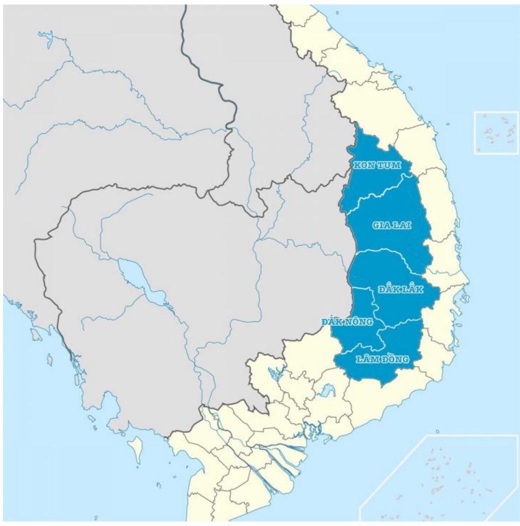

Supplementary Figure S1: Vietnamese Highland *Pygathrix Cinerea* in Tay Nguyen Institute for Scientific Research, Academy of Science and Technology. The dark blue is the highland of Vietnam.
