# Supplementary material for: Characterization of the Complete Mitochondrial Genome of the Central Highland Grey-Shanked Douc Langur (Pygathrix cinerea), a Critically Endangered Species Endemic to Vietnam (Mammalia: Primates)
Source: Curr Issues Mol Biol. 2024 Sep 6;46(9):9928–47. doi: 10.3390/cimb46090592 (PMC11430490; doi:10.3390/cimb46090592)
Supplement: Supplementary file 1 [file cimb-46-00592-s001.zip › Supplementary data 2.pdf]

Supplementary 2: Complete mtDNA genome of Vietnamese Pygathrix Cinerea

TATCTTTTAGGTGTGCATAACTCAACTGTCGTTTTCTCAACTAATAAAAATTTATTTCACA
CACCACCCTTGACACCTCCACCCACCTCTTCTTTCCCATAATCCCCAAAGATGGTATCA
TAATACCCATTATTCAGTTTATGTAGCTTAACACCTCCAAAGCAAGACACTGAAAATGCC
TAGACGGGCTCACAGACCCCATAAACAAACAGGTTTGGTCCCGGCCTTTCTATTAGCTT
TTTAGCAAAATTACACATGCAAGCATCCCCGCTCCAGTGAAGATACCCTATAAATCACG
ACGATTAAGAGGAGTAAGTATCAAGCACGCTTAATGCAGCTCAAGACACTTTGCTTAGC
CACACCCCCACGGGAAACAGCAGTGACTAATATTTAGCAATAAACGAAAGTTTAACTAA
GCTATGCTACTAAGGGTTGGTCAATTTCGTGCCAGCCACCGCGGCCATACGATTGACC
CTAGTTAATAGACCTCGGCGTAAAGGGTGTTTTAGATAAATTTAATTAAATAAAGCTAAA
CTCTTTCTAAACTGTAAAATCCTAGCTAATGTAAAATAGACTACGAAAGTGGCTTTAAAG
CTTCTGAATACACAACAGCTAAGACTCAAACTGGGATTAGATACCCCACTATGCTTAGC
CCTAAACTTTAATAGTTAAAAATAACAAAACTATTCGCCAGAACACTACAAGCAACAGCT
TAAAATTTAAAGGACTTGGCGGTGCTCCATATCCCCCTAGAGGAGCCTGTTCTATAATC
GATAAACCCCGATTCACCTCACCACCTCTTGCTAAGCCTATATACCGCCATCTTCAGCA
AACCTCAACAAGAGATATAAAGTAAGCACAAACGCCCACGCAAAAACGTTAGGTCAAGG
TGTAGCTTATGAGCTGGAAGAAATGGGCTACATTTTCTACCCTAGAAAACCCCACGATA
GCTCTTATGAAACTTAAGAGTCCAAGGAGGATTTAGTAGTAAACTAAGAATAGAGTGCTT
AGTTGAACTAGGCCATGAAGCACGCACACACCGCCCGTCGCTCTCCTCAAATATAATTA
GAACTAACTTAACTAAATATTTCTGCATTCATACAGAGGAGATAAGTCGTAACATGGTAA
GTGTACTGGAAAGTGCACTTGGATAAATCAAGGCATAGCTCAACACAAAGCATCCAGCT
TACACCCGGAAGATATCGCTACCTGATTGCCTTGAGCCAATACTAGCTCAACAAACCTA
CCAATACTACTATTAAATTAATATATTATTAAACCATTCACAAGTATAAAAGTATAGGAGA
TAGAAATTTACTCTGACGCTATAGATGCAGTACCGCAAGGGAAAGATGAAAAAAGCAAC
CAAGCATAAAACAGCAAGGACTCACCCCTATACCTTTTGCATAATGAACTAGCTAGAGA
CCACTTCGCAAAGAGAACTAAAGCCAAGTACCCCGAAACCAGACGAGCTACCCAAAAA
CAGCTAAAAGAGCACACCCGTCTATGTAGCAAAATAGTGGGAAGATTTCTGGGTAGAG
GTGATACGCCTACCGAGCCTGGAGATAGCTGGTTATCCAAGATAGAATTTTAGTTCAAC
CTTAAGTTTACTTACAGAACTACCAATCCTCCTGTAAACTTAACTGTTAGTCTAAAGAGG
GACAGCTCTTTAGACATCAGGAAAAAACCTTATGTAGAGAGTAAAAAATTTCCAAACTCA
TAGTTGGCCTAAAAGCAGCCATCAATTAAGAAAGCGTTTAAGCTCAACGCAATGTCTAA
AAAAATATCAAACACTTTACTGAACTCCTCATATCACATTGGATTAATCTATTATTTCATA
GAAGTAATAATGCTAGCATTAGTAACGTGAATAAATTCTCCAGTGCATAAGCCTAAATCG
GATCGAAACCTTCACCGATATTTGACAGTCTAATGTTATAACCACAAACAAGCCAATATT
ATATATACTGTTAACCCAACACAGGCATGCCCTAAAGGAAAGGTTAAAAAAAGTAAAAG
GAACTCGGCAAACTCAACCCCGCCTGTTTACCAAAAACATCACCTCTAGCATTACCAGT
ATTAGAGGCACTGCCTGCCCAGTGACATATGTTTAACGGCCGCGGTACCCTGACCGTG
CAAAGGTAGCATAATCATTTGTTCTTTAAATAGGGACTTGTATGAATGGCAACACGAGG
GTTTAACTGTCTCTTACTTTCAACCAGTAAAATTGACCTGTCCGTGAAGAGGCGGACAT
AAAATAATAAGACGAGAAGACCCTGTGGAGCTTCAATTTACTAGTACAACCCATTATTAA
AATAAATCTAAGGACCTAACATACCCTGCCCCTGTACTAGAAATTTTGGTTGGGGTGAC
CTCGGAGCATAATTAAACCTCCGAACGAACTACGCCAAGGCCATACAAGTCAAAGCAGT
CTAATATCTAAATTGATCCAATAACTTGACCAACGGAACAAGTTACCCCAGGGATAACA
GCGCAATCCTATTCCAGAGTCCATATCGACAATAGGGTTTACGACCTCGATGTTGGATC
AGGACATCCTAATGGTGCAGCAGCTATCAAGGGTTCGTTTGTTCAACGATTAAAGTCCT
ACGTGATCTGAGTTCAGACCGGAGCAATCCAGGTCGGTTTCTATCTATTCTATATTTCTC
CCTGTACGAAAGGACAAGAGAAATAGGGCCTACTTCACATAAGCGCCCTCCTCCCATAA
ATGACCTAGTCTCAATTTAGCAAGAAATTACACACACACAACCCAAGAACAGGGATTGT
TAAGATGGCAGAGCCCGGTAATTGCATAAAATTTAAGACTTTATAATCAGAGGTTCAACT
CCTCTTCTTAGCACTATGTTCACAATAAATCTTCTACTCATTATCCTACCCACTATAGCTG

CCATAGCATTTCTTACACTTACTGAACGAAAACTATTAGGCTATATACAACTACGCAAAG
GACCCAACATCGTGGGTCCTTATGGACTACTACAACCCTTTGCCGATGCAATAAAACTC
TTCACCAAAGAACCCTTAAAACCCTCAACATCCACTACCACCCTATATATTATTGCACCC
GCCCTAGCCTTTTCTATTGCCCTTCTCCTGTGAGTACCTCTTCCCATACCCAATTCCCTA
ATTAATCTTAATCTAGGACTTCTATTTATCCTAGCTACATCTAGTCTAGCTGTTTACTCCA
TTTTATGATCAGGATGAGCATCCAACTCAAACTACGCATTAATCGGAGCGCTACGAGCA
GTCGCCCAAACAATTTCATACGAGGTAACTCTCGCCATCATTATACTATCAGTTCTACTA
ATAAGTGGCTCATTCAACCTCCACGCACTCATTACAACACAAGAACACCTCTGACTTCT
CCTACCATCATGACCTTTAACCATAATATGATTCACCTCCACACTAGCAGAAACCAATCG
AGCCCCCTTCGACCTCACAGAAGGAGAATCAGAACTAGTATCAGGCTTCAATATCGAGT
ACGCCGCAGGTCCATTCGCTCTTTTCTTCATAGCCGAATATATAAATATTATTATAATAAA
TGCCCTAACAGCCACAATTTTTCTAGGGACACTATACCCAATCCACTCACCAGAACTATT
CACAACATGCTTTGTTACAAAAACTCTTCTCCTAACCTCCTTATTCCTATGGATTCGAGC
AACCTACCCCCGATTCCGTTATGATCAACTCATACACTTACTATGAAAAAATTTCCTTCC
TCTCACATTAGCACTCCTCATATGATATATCTCAACTCTTATTATAACCTCTGGCATCCC
CCCTCAAAGCTAGAAATATGTCTGACAAAAGAATTACTTTGATAGAGTAAATAATAGAGG
TATTCAACCCTCTTATTTCTAGAATTATAGGTATTGAACCTACTCCTGAGAATCCAAATCT
CTCCGTGCTACCTATTACACCTCATTCTAAGTAAGGTCAGCTAAATAAGCTATCGGGCC
CATACCCCGAAAATGTTGGTTATACCCTTCCCGTACTAATTAATCCACTAGCCCAACTTG
TTATCTACTCTACCATAATCATAGGCACCCTTATTACATCACTAAGTTCCCACTGATTTCT
AGCCTGAACCGGCCTAGAGATAAATATACTAGCTTTTACCTCAATCCTAATTAAAAAAGC
AAACATTCGCTCCACAGAAGCTGCTACCAAGTATTTCCTTACACAAGCCACCGCATCTA
TAATTCTCATAATAGCAATCATGTATAACAACCTACTCTCAGGACAATGAACCCTGATAA
ACAATCCCAATCAACTCTCATCCTTAATCATAACAATAGCACTCGCTATAAAATTAGGAA
TAACCCCCTTCCACTTTTGAGTTCCAGAAGTCACCCAAGGAACACCCTTAATATCCGGC
CTGCTTCTCCTCACATGACAAAAACTAGCCCCTATCTCAATTATATATCAAATTTATCCAT
CAATTAACACAAGTATTCTTATAACCCTATCAACCCTATCCATCATAGCAGGCAGTTGAG
GGGGTCTCAATCAAACACAACTACGAAAAATTCTAGCATACTCTTCAATCACACACATAG
GCTGAATAATAATAACAATAACGTATAACCCAAATATTACAATCTTCTATCTGCTCATGTA
CATCATTATAACAAGCACTGCATTCCTAGCCCTGAACCTAAACTCAAATACCACCACCCT
AATACTATCACGCACCTGAAACAAACTAACCTGACTAATACCATTAATACTACTTACCCT
CCTATCCATAGGAGGTCTACCTCCACTGACCGGCTTTCTACCTAAATGGATAACAATTC
AAGAACTTACAAAAAATAGCAACTTTATCATACCCTCTATCATAATCACCATAACTTTACT
CAATCTATATTTCTATTTACGTTTAACTTATATTACTTCCATAACACTACTCCCTACGTCTA
ATAACACAAAAATAAAATGACAGTTCGAAAATACAAAGCCTACACCCCTTTTCCCCCCAC
TAATTATTTTTACAACCCTTCTCTTACCAATATCGCCAACAGTTCTAACCCTATTCTAGAA
ATTTAGGTTAAACCAGACCAAAAGCCTTCAAAGCTTTCAGTAAGTTAACATGCTTAATTT
CTGAAACACATAAGGACTGCAGTACTATACCCTGCATCAACTGAACGCAAATCAATCAC
TTTAATTAAGCTAAGCCCTTACTAGATCAATGGGATTCAAACCCACAAAAACTTAGTTAA
CAGCTAAATACCCTAATCAACTGGCTTTAATCTACTTCTCCCGCCGCAGGGAAAAAAAG
GCGGGAGAAGCCCCGGCAGAAATAAAACTGCTCCCTTGAATTTGCAATTCAACATGATA
ATCACCTCGGGGCTGGTAAAAAGAGGGTTCAACCTCTGTACTTAGGTTTACAGCCCAAT
GCCTACTCAGCCATTTTACCTATGCTCATCAACCGCTGGTTATTCTCTACAAATCACAAG
GACATTGGAACTTTGTATTTATTATTTGGTGCATGAGCTGGAACCACAGGTATAGCTATA
AGTCTCCTTATTCGAGCTGAACTAGGCCAGCCCGGCAACCTACTAGGCAACGACCATA
TTTATAATGTTATTGTTACAGCCCATGCATTTGTTATAATTTTCTTCATGGTTATACCAATT
ATAATCGGGGGCTTCGGAAACTGACTAGTTCCCTTAATAATTGGCGCTCCTGACATAGC
ATTCCCCCGCCTAAATAATATAAGCTTCTGACTTCTCCCGCCATCTTTCCTACTTCTTCT
CGCATCAGCAATAGTAGAGGCTGGCGCAGGAACAGGCTGAACAGTCTATCCTCCTCTA
GCAGGAAATTTTTCCCACCCAGGAGCTTCTGTAGACTTAACTATTTTTTCACTCCACCTA
GCAGGTATTTCCTCTATCTTAGGAGCTATTAATTTTATTACTACTATTATTAACATAAAAC

CCCCTGCCATGTCTCAGTATCAGACACCCCTATTTGTTTGATCCGTCCTAATTACAGCA
GTCTTACTGCTTCTATCCTTACCTGTATTAGCTGCGGGCATTACAATGCTATTAACAGAC
CGTAATCTCAACACTACCTTCTTTGACCCCGCCGGAGGAGGAGACCCAATCTTATATCA
ACACTTATTCTGATTTTTCGGTCACCCTGAGGTTTATATTCTTATCTTACCTGGGTTTGG
AATGATCTCCCACATTGTAACATATTATTCCGGAAAAAAGGAACCATTCGGATATATAGG
CATAGTCTGAGCTATAGTATCAATTGGGTTTTTAGGCTTTATCGTATGAGCTCACCATAT
ATTTACTGTTGGCATAGACGTAGACACACGAGCCTATTTCACCTCCGCCACCATAATTAT
TGCAATCCCAACTGGCGTTAAAGTCTTTAGCTGACTAGCTACACTACATGGAGGAAATA
TCAAATGATCCCCTGCAATACTCTGAGCCCTAGGCTTTATTTTCCTCTTCACCGTAGGA
GGCTTAACTGGTATTGTACTAGCAAACTCATCACTAGATATCGTACTACATGATACATAC
TATGTGGTAGCCCACTTCCACTACGTTTTGTCAATAGGAGCCGTCTTTGCTATCATAGG
GGGTTTTATCCACTGATTTCCACTATTCTCAGGATACGCCCTAGACGAAATTTGTGCCAA
AGCCCACTTTATTATTATATTTGTAGGCGTAAATTTAACTTTCTTTCCGCAGCATTTTCTT
GGTTTGTCCGGAATACCTCGACGTTATTCCGATTATCCTGATGCTTACACCACATGAAAT
GTTGTATCATCTATAGGCTCCTTCATCTCCCTAGTAGCTATATTACTAATAATCTATATAA
TCTGAGAAGCTTTCGCCTCAAAACGTAAAGTCCTATTTATTGAACAACCTACCTACAACC
TAGAATGATTGCACGGCTCTCCACCACCATATCATACATTCGACGAACCAACATTCATTA
AGGTTAAATAAAAAAGGAAGGAATCGAACCTCCTGAGATTGGTTTCAAGCCAATCCTAT
AACCTCTATAACTTTTTCAATAAGATATTAGAAAAAATTATTTCATGGTTTTGTCAAAGCT
AAATTATAGGATACACCCTATATATCTTATATGCCTCACCCAGTTCAACTAGGCCTACAA
GATGCCACATCCCCTATCATAGAAGAATTAATTGCCTTCCACGACCATGCCTTTATAATT
GTAGCTATGATCAGCTTTTTAGTCTTATACGTCTTATCTTCAGTACTCACAACAAAATTAA
CTAACACCAATATTACAGATGCTCAAGAAATAGAAACTATTTGGACCATCTTACCAGCAA
TTATCTTAATCTTAATTGCTCTGCCATCCCTACGTATTCTCTACTTAACGGATGAGGTTAA
TAATCCTTCATTTACTATTAAATCAATCGGACATCAGTGATACTGAACTTATGAGTATACA
GATTATGGGGGCTTAATCTTTAATTCCTATATACTCCCTCCACTATTCTTAAACCCAGGG
GACCTTCGACTTCTGGAAGTTGATAATCGAGTGGTACTCCCAATTGAAGCTCCTGTTCG
CATAATAATTACATCTCAAGACGTCTTACACTCCTGAACTATTCCTACGCTAGGATTAAA
AACAGATGCAATTCCCGGACGCTTAAATCAAACCACATTCACTGCCACACGACCAGGTG
TCTACTACGGACAGTGCTCAGAAATTTGCGGCGCTAATCACAGTTTTATACCAATTGTTG
CAGAACTAATTCCACTAAAAATTTTTGAAATAGGGCCTGTATTCACCCTGTAGATATACT
AAAGCGTTTAATTAAAGTATTTCTTAAGTACTAAACTCACTGCATAGCTGCCACAGCATT
AACCTTTTAAGTTAAAGACTGAGAAAATATTCTTCTCTGCAGTGAAATGCCTCAACTAAA
TACATCTACATGGCTAATTACTATTATAACCATGCTACCCGCACTATATCTTATTATACAA
CTAAAACTATTAAATATAGTCTACTATTTTCCCCCGTCACAAAAAGTCTCTAGTACGCAA
ATATTTAACAACCCCTGACAACTAAAATGAACGAAAATTTATTTACCTCATTTACACACCC
AACACTCCTAGGGCTACCAGCCGTAGTACCTATTATTCTATTTCCTACATTACTACTTCC
AACCTCAAAATATCTTATTAATAATCGACTAATTACTATTCAACAAAATCTAATTCAGCTA
ATCGTAAAACAAATAATAATAATTCATAATATTAAAGGACAAACCTGGTCTCTAATACTAA
TATCCCTAATCATTTTTATTGCCACAACTAACCTTCTCGGACTCTTACCCCACTCATTTAC
ACCTACCGCCCAACTATCAATAAATCTGGCTATAGCAATCCCTCTATGAGCAGGTACAG
TAATCACAGGCCTTCGCTTCAAAACTAAAAGCTCCTTAGCGCACTTTTTACCACAAGGTA
CACCCACACTACTTATCCCCATATTAGTAGTTATTGAGACCATCAGTTTATTTATTCAACC
AGTAGCCCTTGCTGTACGCCTAACCGCCAATATTACAGCAGGACATCTACTTATGCACC
TAATTGGAAGTGCTACGCTAGTACTATCAACCATCAGCCTCTCTATAACCTCACTAACTT
TAGTGCTTCTAGTATTATTAACAATTCTAGAAATAGCAGTCGCCCTAATTCAAGCCTACG
TTTTCACACTATTGGTAAGCCTTTATCTACATGACAACACTTAATGACACACCAAACCCA
CCCCTATCACATAGTTAAACCCAGTCCATGACCACTGACAGGAGCCCTGTCAGCTCTCC
TAATAACATCTGGCCTAATTATATGATTCCACTTTTATTCTACAACCCTATTAACCCTAGG
TCTACTAACCAATATGTTAACCATATACCAATGATGGCGTGATATCATCCGAGAAAGCAC
CTATCAAGGTCACCATACAACACCAGTTCAAAAAGGCCTCCGCTACGGAATAGTATTAT

TTATCATCTCAGAGGTTTTCTTCTTTGCTGGCTTCTTCTGAGCTTTTTATCACTCAAGCCT
GGCCCCGACTCCACACCTAGGAGGACACTGACCACCTACAGGTATTATTCCCCTAAAC
CCCCTAGAAGTACCCCTTCTAAATACCTCCGTACTACTTGCATCAGGGGTTACAATCAC
TTGAGCTCACCACAGCTTAATAGAAAATAACCGAAAACAAATAATTCAAGCCCTATCAAC
TACAATTTTATTAGGCATTTATTTCACCCTACTACAAATATCAGAGTACTATGAAGCACCC
TTTACCATCTCTGATGGAATCTATGGTTCAACATTCTTTGTTGCTACCGGCTTTCATGGA
CTTCATGTTATCATTGGATCTACATTTCTTACTACCTGTCTTATTCGTCAGCTATCGTACC
ATTTTACATCAAGTCACCATTTTGGCTTTGAAGCTGCCGCCTGATATTGACATTTCGTAG
ATGTCGTCTGACTCTTTCTCTATATCTCCATTTACTGATGAGGTTCCTATCCTTTTAGTAT
AACTAGTACAGCCGACTTCCAATCAGTTAGTTTCGATAATATCGAAAAAGGATAATTAAT
CTAATACTAACTCTAATAATTAATACCTCCTTAACCATCTTGCTAATAATTATTATATTTTG
GCTACCCCAACTCAACTCTTATGTAGAAAAAGCCAACCCCTACGAATGCGGGTTTGACC
CACTAAACTCTGCTCGCATTCCCTTTTCCATAAAATTCTTCCTAGTCGCTATTACCTTCCT
GCTGTTTGATTTAGAAATCGCCTTGCTATTGCCCTTGCCATGAGCCCTCCAAACAACAA
ACCTTCCCGTAATGATCAAGTCATCAATTACATTAATTATTATCTTAACCCTCAGCCTGG
CCTATGAATGAACTCAAAAAGGTTTAGATTGAACTGAATTGGTAAGTAGTTTAAGTAAAA
CAAATGATTTCGACTCATTAGATTATGGTAATCATACTAACCAAATGTCCATTATTTATAT
AAACATTATATTAGCATTCACTACCTCACTCCTGGGCATATTAATCTATCGCTCACATCT
AATGTCATCCCTACTATGCCTAGAAGGAATAATACTTTCACTGTTTATCATAAGCACTCT
TACTGCATTAAACACTCACTTTCCCCTCGCCAATATAGTACCTATTGCCTTACTAGTATTT
GCCGCTTGCGAGGCAGCAGTGGGCCTTGCCCTATTAATTTCAATCTCAAACACATACG
GCTTAGACCATATCCAGAACCTAAACTTACTTCAATGTTAAAAATAATTTTCCCTACAATG
ATACTATTACCAACAACATGATTTTCCAAAAACAACCTAGTCTGAATTAACCTAACTACAC
ATAGCTTAATAATCAGCCTCATTCCCCTTATATTCTTCAATCAAATCAATAATAACCTTAT
TAGCCACTCGACCTATTTATCTTCAGATCCATTAACAACACCTCTTCTAATACTGACAGC
CTGACTCCTGCCCCTCATAATTATAGCAAGCCAATATCACCTACACAATGAAAGTCCCC
TGCGAAAAAAACTTTACCTCTCCATAATAATCTTCCTACAAATCTCCCTAATCATAACATT
TATAGCCACAGAGTTAATCTTATTCTACATCCTATTTGAAACAACCCTTATCCCCACCCT
AATTATTATCACCCGATGAGGTAACCAAGCAGAACGCCTCAACGCAAGCACATATTTCT
TATTTTATACATTAGCTGGCTCCCTACCTCTGCTAATTATATTAATTTATACACATAACAA
ATTAGGCTCATTAAATATTCCACTACTAACACTCATAGCCCAAAAACTAACAACCTCTTG
ATCCCATAATTTAGCTTGACTAGCATGCATAATGGCTTTTATAGTAAAAATACCCTTATAT
GGCCTACACCTATGACTCCCTAAAGCCCATGTTGAGGCTCCCATTGCCGGGTCAATAG
TTCTTGCCGCCGTGCTCTTAAAATTAGGCGGCTATGGCATAATACGACTTACACCAATT
CTCAACCCACTAACAGAACATATAGCCTACCCTTTTCTTATACTATCCTTATGGGGCATA
ATTATAACTAGCTCAACCTGTCTCCGACAAACAGATTTAAAATCGCTCATTGCATACTCC
TCTGTAAGCCACATAGCCCTTGTAATCATAGCCTCCCTCATTCAAACCCCCTGAAGCTTT
ACTGGCGCTATCATCCTTATAATTGCCCACGGACTTACCTCATCTATATTATTCTGCTTA
GCAAACTCAAATTACGAACGAACCCACAGCCGCATTATATTACTTTCTCGAGGACTTCA
AAGTCTACTTCCACTAATAGCCTTCTGATGATTTGTAGCAAACCTCACCAATCTAGCTCT
ACCTCCCTCCATTAACTTAATCGGGGAGTTATTAGTAGTGACATCCTCATTTTCTTGGTC
ACATATTACCATTATCTTCACAGGACTAAACATATTAATTACTGCTCTATATTCCCTACAC
ATATTCATTACAACACAACGAGGAACACTCACCTCCCATATTATTAACATAAAACCCTCT
TTTACACGAGAAAACATGCTAATATTTATACATATATCTCCTATTATTCTTCTAACCCTTA
ATCCTAGCATTATTATAGGCTTCACCCCTTGTAAATATAGTTTAATTAAAACATTAGATTG
TGAATCTAAATATAGAAACCTACCACTTCTTATTTACCGAGAAAGATTGCAAGGACTGCT
AATCCATGCCCCCGTATTTAATAAAACGGCTATCTCAACTTTTAAAGGATAACAGCTGTC
CATTGGTCTTAGGAACCAAAAATATTGGTGCAACTCCAAATAAAAGTAATAATAATGCAC
ACCTCCATTTTTATACTAGCCCTAACCCCCTTAATCTTTCCGATTATTATTACCCTTATTA
GCCCCAATAAAAATAATATATACCCCAACTATGTAAAAACAACTATGATATTTACCTTTAC
TATTAGTCTCATCCCCACAACCATATATACTTTCCTAGGTCAAGATACAATCATATCAAC

CTGACATTGAATAACTATTCAATCACTAGAAATTACACTAAGTTTTAAATTGGACTATTAC
TCCGTAATATTTACCCCAATTGCACTATTTATTACTTGGTGCATTATAGAATTCTCACTAT
GATACATAGACTCAGACCCAAACATTAACCAATTCTTCAAATATCTTCTCATCTTCCTTAT
TACCATGCTAATTTTAGTTACCGCTAACAACCTCTTCCAGCTCTTTATTGGGTGAGAAGG
TGTAGGAATTATATCATTTCTACTAATCGGCTGATGATACGCTCGAACAGACGCTAACAC
AGCAGCCATTCAAGCAATTCTGTATAACCGCATTGGTGATATTGGTTTCATTCTAGCCAT
AATATGGTTTCTCCTCCATTATAACTCATGAGACTTACAACAAATATTTATCCTAGATCCC
AACCCCGATCTACTTCCATTAGTGGGTCTACTATTAGCAGCAACAGGAAAATCAGCCCA
ACTCGGCCTCCATCCCTGATTACCCTCGGCTATAGAAGGCCCAACTCCAGTATCAGCC
CTACTTCACTCCAGTACTATAGTAGTAGCTGGGGTTTTCTTACTTATTCGCTTCCACCCA
CCAATAGAAAATAATACAACAATTCAAAGTCTTACACTATGCCTAGGAGCTATTACTACC
ATATTCATAGCAATCTGCGCCCTAACACAAAATGACATTAAAAAAATTGTAGCCTTCTCT
ACCTCAAGTCAACTGGGACTTATAATAGTTACTATTGGTATTAATCAACCGCACCTAGCA
TTTCTACATATCTGTACTCATGCCTTTTTCAAGGCTATACTATTTATCTGCTCTGGGTCTA
TAATTCATAACCTAAATAATGAACAAGACATCCGAAAAATAGGAGGACTATTTAAAACAA
TGCCCCTCACCTCAACTTCCCTGATAATCGGTAGCCTAGCACTCACAGGCATACCTTTT
CTTACAGGTTATTACTCCAAAGACCTCATCATCGAAACCGCAAACACATCATACACCAAC
GCCTGGGCCCTGTGTATTACTCTTATCGCTACCTCTATAACAAGCGCCTACAGCACCCG
AACTATTATTCTCACACTAACAGGATCACCTCGTTTTTCAACTTCCGTATATATTAATGAG
AACAACCCAACCCTACTAAACCCAATAAAACGCCTAGCAGCAGGTAGTCTACTCGCAGG
ATTTTTCATCGTCAACAACATCTCTCCGACTACAGTTCCTCAATTAACAATACCTTATCAC
CTGAAACTCCTAGCCTTATGCGTAACCACCCTAGGCTTCTTAACAGCCCTAGATCTGAC
TCTTATAACTAACAGTCTCAAAATAAATACCCCATCGCACATATTCAAATTCTCCAATATA
CTAGGATATTTTCCCATTACAATTCACCGAACAGTTCCCTACCAAAACCTAACCATAAGC
CAAAACCTAGCCTTCCTATTACTAGACTTACTCTGACTAGAGAAATCAATACCTAAAACA
ATTTCACACACCCATATTATTACAGCCATCACCTCAACCACCCAAAAAGGCATAATCAAG
CTATATTTCCTCTCTTTTCTTATTTCCCTCACACTAATCCCACTTTTAATTATATAATCTAT
TACCCCGAGTAATTTCAATAACAATATAAACACCAACAAATAATGTTCAACCAACAACTA
CGACCAACCAACGCCCATAATCATACAAAGCACCCGCACCAATAGAATCCTCACGAATC
AACCCCGACCCCTCCCCCTCAAAAATCACCCAACTCCCTATATTATCCAAATTAATTATC
ACTACCAACTCATTATAATCTATAACCCACAAAACTAAATATACCTCCATTGCCAATCCAA
CCAAAAAACTCCCCAAAACCTCAAATCCTGAAACCCATGCTTCAGGATATTCTTCAATAG
CCATCGCAGTAGTATAACCAAAGACAACCATTATACCCCCCAGATAAATCAAAAACATTA
TTAAACCTATATAAGTACCCCCATAACTTAAAATAATAGCACAACCAATCACACCACTAA
CAACCAATGCTAAACCCCCATAAATAGGAGAAGGCTTAGAAGAAAAGCCCACAAAACCC
ATAACTAATAATACGCTTAATAAAAATAAAATATACGACATTGTTTCCACATGGACTCTAA
CCATGATTAATGATATGAAAAACCATCGTTGTATTTCAACTATAAAAACACTAATGATCCC
CGTACGCAAATCTAATCCAATTATAAAATTAATTAATCACTCCCTCATTGATCTACCAACC
CCATCAAACATCTCAGCATGATGAAACTTTGGTTCCCTTTTAGCAACCTGCTTAATTTTA
CAAATCATCACAGGTCTATTCCTAGCAATACATTACTCACCTAGCACCTCCTCAGCCTTC
TCCTCAATCGCCCATATCACTCGAGACGTAAACTACGGCTGAATTATCCGCTACCTTCA
TGCCAATGGTGCCTCCATATTCTTTATCTGCCTATTCCTACACGTAGGTCGAGGGTTATA
TTATGGCTCATTCCTTCTTCTTGAAACTTGAAACATTGGCATTGCACTATTACTTATAGTT
ATAGCAACGGCCTTTATAGGCTATGTACTCCCATGAGGACAAATATCATTTTGAGGTGC
TACAGTAATTACAAATTTATTATCCGCAATCCCATACATCGGAACAAATCTCGTTCAATG
AGTATGAGGTGGATATTCTATCGATAATCCAACCCTTACCCGATTCTTCACTCTCCACTT
TACCCTGCCTTTCATTATCACAGCCTTTACAGTTCTACATCTACTTTTCCTACACGAAAC
AGGATCTAATAATCCATGCGGAATTCCCTCAAACTCCGACAAAATCCCCTTCCACCCCT
ACTACACAATCAAAGATATGCTAGGCCTAGTCCTCCTTATTCTTCCCCTAATAACTCTAG
TATTATTTTCACCTGACCTTTTAGGTGACCCAGACAACTATACACCAGCTAATCCACTAA
ACACCCCACCACACATCAAACCAGAATGGTACTTCTTATTCGCATATGCAATCCTACGAT

CTGTACCTAACAAATTGGGAGGCGTACTAGCACTTCTTATATCCATTCTTATCCTAATAA
TTATCCCCATACTTCACAAATCCAAACAACAAAGCATAATATTCCGCCCATTCAGCCAAT
TTACACTATGATTATTAATCACAGTTCTATTAACCCTAACCTGAATTGGAAGCCAACCAG
TAAACCAACCCTTTATTATAATTGGACAGGTAGCATCTATAATATATTTCACTACAATTCT
GATCTTAATACCACTAGCCTCTATAATCGAAAACAATCTCCTCAAATGAACCTGCCCTTG
TAGTATAGACTAATACACCGGTCTTGTAAACCGGAGACGGATACCTTTCCCCAGGGCAA
CTCAGAAAGAAAGCATTTAACTCCTCCACCAATACCCAAAACTGGCATTCTATTTAAACT
ACTTTCTGCATTCTAAGGAGGTATAACCTTTAGAGAATAATTTAGTACAATCTAATTTTAT
ATGTCCTTATGTAATTCGTGCATTACTGCTAGTCACCATGGATATTATATAGTACTATAAA
TGTTTTACCGTCCATAGGACATAAAATTACATATTTACTGGCAGTTATATCTAGGACATG
CTTATAAGCAAGTACTCTAATAGAATATCTGACTATAACACATACCATTCACATATCCAAA
TCCCGTGGTCACACCCATTGGAATACCAACCAATAGCGATTAATCCATTATCGTACATA
GTACATAATATTCTTTACCGGACATAGCACATCGCAGTCGAGCATCCCTAAACCAATCC
TCGTCAACACGGATATTCCCGTCAGTTAGGTGTCCCTTGATCACCATCCTCCGTGAAAT
CAATATCCCGCACAAGAGTGCTACTCTCCTCGCCCCGGGCCCATAACCTGTGGGGGTA
GCTACACACGACGTCAATGGACATCTGGTTCTTACCTCAGGGCCATACCGCCAAGATC
GCCCACACGTTCCCCTTAAATAAGACATCTCGATGGATCACGGGTCTATCACCCTATTA
ACCAGTCACGGGAGCTCTCCATGCATTTGGTATCTTTTATCTCTGGTCTGCACGCGACC
CCATTGCAGTATGCTGGTCTCGCCACAATCAGTCCCGCAGCGCCTGTCTTTGATTCCTA
GCACATACCATTATTAACCGCACCTACGTTCCATATTTTAATCCCGCATGAACCTTATCA
TGGTGTTATTTAATCCATGCTTGTAAGACATACATATAATTAACCGTATACCCCGATCGC
ACCTTGATCCAATTTACAACTATCTCTACTCAAACCCCCCCCCCCCCATATTCGACCG
CACCCTAATAATCCGCCTTTGCCAAACCCCAAAAACAAAAACTTGCAATCTGGTCGAATT
TTACATTTTTTTTT
