# Supplementary material for: Characterization of the Complete Mitochondrial Genome of the Central Highland Grey-Shanked Douc Langur (Pygathrix cinerea), a Critically Endangered Species Endemic to Vietnam (Mammalia: Primates)
Source: Curr Issues Mol Biol. 2024 Sep 6;46(9):9928–47. doi: 10.3390/cimb46090592 (PMC11430490; doi:10.3390/cimb46090592)
Supplement: Supplementary file 1 [file cimb-46-00592-s001.zip › Supplementary data 3.pdf]

Supplementary 3: Read quality (Illumina) of analyzed samples.

Sample		Read total	Base total (bp)	Length (bp)	%GC	%Q30
	Read R1	15,751,624	2,362,743,600	35-151	45.8	91.1
	Read R2	15,751,624	2,362,743,600	35-151	46.1	88.7
