# Supplementary material for: Characterization of the Complete Mitochondrial Genome of the Central Highland Grey-Shanked Douc Langur (Pygathrix cinerea), a Critically Endangered Species Endemic to Vietnam (Mammalia: Primates)
Source: Curr Issues Mol Biol. 2024 Sep 6;46(9):9928–47. doi: 10.3390/cimb46090592 (PMC11430490; doi:10.3390/cimb46090592)
Supplement: Supplementary file 1 [file cimb-46-00592-s001.zip › Supplementary data 4.pdf]

Supplementary 4: De novo assembly result

Number of contig	1
Number of loop	1
% GC	38.9
Length of contig	16,656
Average coverage of read assembly	63.6
